# Improved Chiral Separation of (R,S)-Goitrin by SFC: An Application in Traditional Chinese Medicine

**DOI:** 10.1155/2016/5782942

**Published:** 2016-02-21

**Authors:** Lixing Nie, Zhong Dai, Shuangcheng Ma

**Affiliations:** National Institutes for Food and Drug Control, China Food and Drug Administration, 2 Tiantan Xili, Beijing 100050, China

## Abstract

Like chemical drugs, research and development of herbal medicine also have a need to resolve enantiomers. To help illustrating the antiviral bioactivity of Isatidis Radix, a widely used traditional Chinese medicine (TCM), supercritical fluid chromatography (SFC) was used for analytical and preparative separation of (R,S)-goitrin, which was reported as the active ingredient of the herbal. Improved resolution was achieved on Chiralpak IC column, using acetonitrile as the organic modifier, representing a tenfold increase in speed, compared to the previous normal phase HPLC (NPLC) method. The newly developed chromatographic method was validated in terms of linearity, precision, limit of detection (LOD), and limit of quantitation (LOQ). Scale-up purification of (R)-goitrin and (S)-goitrin was performed on a preparative column with >90% total recovery. The absolute stereochemical assignment of the purified isomers was determined through optical rotation study. This attempt explored SFC's application in chiral research of traditional Chinese medicine.

## 1. Introduction 

**Figure 1:**
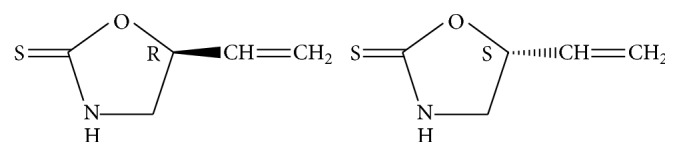
Chemical structures of (+)-(R)-goitrin (epigoitrin) and (−)-(S)-goitrin (goitrin).

As a fundamental characteristic of chemical substances, the phenomenon of chirality also exists in natural products. For instance, Isatidis Radix (root of Isatis indigotica Fort.) is one of the most commonly used traditional Chinese medicines in China, whose indications include pestilence and seasonal toxin, fever and sore throat, macula and papule caused by warm toxin, mumps, scarlatina, erysipelas facialis, erysipelas, and swelling abscess [1]. Antiviral activity of this herb has drawn great attention due to its successful application in epidemics like severe acute respiratory syndrome (SARS) and avian influenza [2–4]. Results of in vivo studies indicated that epigoitrin ((+)-(R)-goitrin) was the therapeutic agent [5–7]. However, neither of the epigoitrin used in the studies was optically pure when the source of the material used in their reports was investigated. By testing their optical rotation values, it was found that they were mixtures of unequal amounts of (+)-(R)-goitrin (epigoitrin) and (−)-(S)-goitrin (goitrin), which could not be resolved by regular C_18_ column. Here, the mixture was named as “(R,S)-goitrin.” To address this issue, a normal phase HPLC (NPLC) based methodology was developed by the authors to separate R-goitrin and S-goitrin and was applied in chiral analysis of Isatidis Radix [8]. The structure of the enantiomers can be seen in Figure 1.

Over the past decades, chiral separation has become an important research topic in the analytical science arsenal [9–11]. Individual enantiomers of chemical drugs may evince very different bioactivities and/or biotoxicities, so do natural products. While one isomer possesses a therapeutic effect, its enantiomer may be inactive and antagonistic or even has reverse effect. With this perspective in mind, it is imperative to investigate pharmacology and toxicology of (R)-goitrin and (S)-goitrin, respectively, which means large amount of resolved enantiomers need to be prepared for tests in vivo and in vitro.

Generally, HPLC is the most frequently used technique for preparative separation [12–15]. But when the established NPLC method was scaled up into a high-throughput way, things became less efficient due to long analytical time (about 30 min), large solvent consumption (especially non-environmentally friendly solvent such as *n*-hexane), and heavy solvent recovery work. Luckily, industrial application of supercritical fluids offered the chemists a new option [16, 17]. Recently supercritical fluid chromatography (SFC) has been viewed as a preferred approach showing advantages in shorter time and lower cost [18–22]. Major part of the solvent used in the mobile phase of SFC is supercritical CO_2_, low viscosity and high diffusivity of which allow higher flow rate than HPLC. This is particularly beneficial when removing solvents after preparative resolution.

In this study, SFC was adopted to develop a more efficient method for separation of (R,S)-goitrin, broadening its application in chiral investigation of TCM. The analytical method was validated and compared to the former work in chromatographic performance. And the preparative method was used in purification of (R)-goitrin and (S)-goitrin.

## 2. Experimental

### 2.1. Chemicals and Reagents

Carbon dioxide (food-grade) was obtained from Chengweifeng Inc. (Beijing, China). All the solvents were HPLC grade and were purchased from Merck (Darmstadt, Germany). (R,S)-goitrin (product number 111753) was from National Institutions for Food and Drug Control (Beijing, China). (R)-goitrin and (S)-goitrin were previously prepared by the NPLC method.

### 2.2. Apparatus

Analytical and preparative SFC separations were performed on a Waters Investigator SFC System controlled by ChromScope software (Waters, Milford, MA, USA). The system consisted of a FDM10 fluid control module, an Alias autosampler, a 10-port Thar columns oven, and a 2998 photodiode array (PDA) detector. Optical rotation of the separated enantiomers was measured by a Rudolph Autopol IV polarimeter.

### 2.3. Chiral Stationary Phases (CSPs)

The Daicel Chiralpak ADH, Chiralpak ASH, Chiralcel ODH, Chiralpak IA, and Chiralpak IC analytical columns (4.6 mm × 250 mm, 5 *μ*m) and the preparative scale Chiralpak IC column (20 mm × 250 mm, 5 *μ*m) were purchased from Daicel Chiral Technologies (Shanghai, China).

### 2.4. SFC Conditions and Sample Preparation

The mobile phase was comprised of carbon dioxide and cosolvent. Analytes were detected at a wavelength of 245 nm. The scan range of PDA was from 190 nm to 380 nm and the compensated reference was set from 270 nm to 380 nm. Injection volumes were 10 *μ*L and 100 *μ*L for analytical and preparative separation, respectively. For analytical SFC, type and ratio of modifier/mixed modifier, column temperature, flow rate, and back pressure were examined to adjust the mobile phase elution strength. For milligram SFC, ratio of modifier and flow rate were reexamined to get the best preparative efficiency. (R,S)-goitrin was prepared with 100% methanol for both analytical and preparative separation.

### 2.5. Method Validation

The proposed analytical method was validated as per the recommendations laid down by International Conference on Harmonization (ICH) guideline [23]. For linearity test, stock standard solution was prepared by dissolving 20.0 mg of (R)-goitrin and (S)-goitrin in 10 mL methanol (2.0 mg/mL), respectively. For precision test, the intraday and interday variation for determination were carried out at three different concentration levels: 0.0003, 0.1, and 2.0 mg/mL. Three replicates were performed for each concentration. The limit of detection (LOD) and limit of quantitation (LOQ) were separately determined at a signal to noise ratio (*S*/*N*) of 3 and 10.

## 3. Results and Discussion

### 3.1. Analytical Method Development

**Figure 2:**
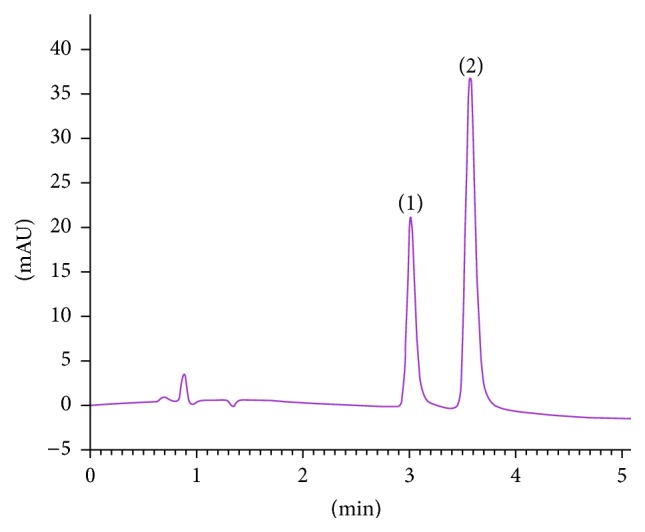
Chiral separation of (R,S)-goitrin using improved SFC method. (1) (S)-goitrin; (2) (R)-goitrin. Chiralpak IC 4.6 × 250 mm column, 5 *μ*m; ACN/CO_2_ (15 : 85, v/v); 3.5 mL/min, 35°C, 100 bar, UV detection 245 nm.

**Figure 3:**
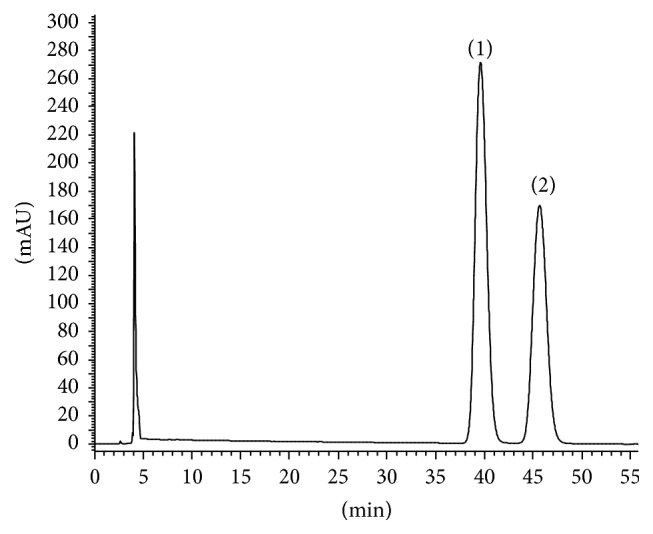
Chiral separation of (R,S)-goitrin using previous NPLC method. (1) (R)-goitrin; (2) (S)-goitrin. Chiralpak IC 4.6 × 250 mm column, 5 *μ*m; *n*-hexane/isopropanol (90 : 10, v/v); 0.8 mL/min, 20°C, UV detection 245 nm.

Initial chiral screening was automated on SFC using 5 different polysaccharide CSPs (Chiralpak ADH, Chiralpak ASH, Chiralcel ODH, Chiralpak IA, and Chiralpak IC) cross-matched with 5 different organic modifiers (MeOH, EtOH, ACN, IPA, and 50 : 50 MeOH/EtOH solvent). For each combination, the percentages of the modifier were set at 5%, 10%, and 20%. Though clear separation of R-goitrin and S-goitrin could be observed either on Chiralcel ODH coupled with MeOH or on Chiralpak IC coupled with ACN, the greatest potential for preparative purification was demonstrated on the latter combination. Therefore, further SFC conditions were optimized on Chiralpak IC column using acetonitrile as cosolvent. Four parameters, namely, modifier concentration, column temperature, flow rate, and back pressure, were chosen as optimization factors. Retention time (Rt) and resolution (Rs) were used as criteria to evaluate the separation quality. As can be found in Tables 1 [Table tab2] [Table tab3]–4, composition of the mobile phase shows dominant influence on Rt and Rs, while the other 3 factors have relatively weaker impact on separation. It is obvious that when less acetonitrile is used, larger resolution will be obtained. But the analysis time will be extended at the same time. Finally, the condition that gave sufficient resolution and relatively shorter analysis time was chosen. At 35°C, 15% ACN (3.5 mL/min), and 100 bar, satisfactory resolution (Rs = 3.75) and quick separation (within 5 min) were achieved. Following optimization, a new chiral analytical method was obtained. The total analysis time represented a tenfold increase in speed, compared to the authors' previous established NPLC method (see Figures 2 and 3). The peaks were identified by running the (R)-goitrin and (S)-goitrin standard. Interestingly, the elution order of (R)-goitrin and (S)-goitrin was reversed on the same column in SFC and HPLC (Figures 2 and 3).

### 3.2. Linearity, Precision, LOD, and LOQ

In the range of 0.0003–2.0 mg/mL, the linearity of calibration graphs and adherence of the system to Beer's law were validated by high value of correlation coefficient. The repeatability of sample application and measurement of peak area were expressed in terms of relative standard deviation (RSD). The RSD values for intraday and interday variation were all below 3.0%. The LOD and LOQ reached ng degree, much lower than those of the NPLC method [8]. This improvement benefited from reference wavelength compensation technique we used in data acquisition, which collects wideband absorbance data in a region where the analytes have minimal or no absorption. The detector calculates the compensation value by averaging the absorbance values within the selected range of wavelengths. The averaged value is then subtracted from the absorbance value. Since the main absorbance (190 nm to 380 nm) includes the reference bands (270 nm to 380 nm), noises from mechanical and thermal noise can be effectively reduced [24]. All the method validation data are summarized in Table 5.

### 3.3. Semipreparative Isolation of the Enantiomers

**Figure 4:**
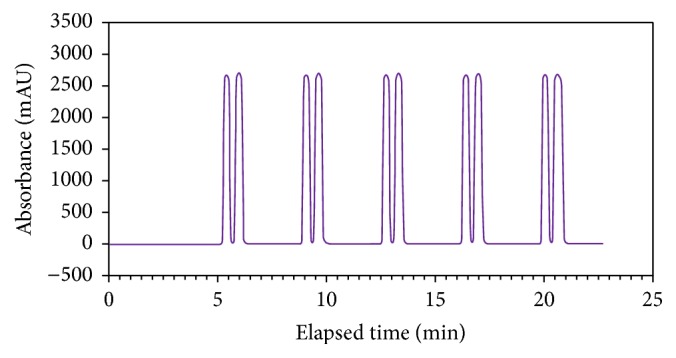
Preparative chiral separation of (R,S)-goitrin. Chiralpak IC 20 × 250 mm column, 5 *μ*m; ACN/CO_2_ (15 : 85, v/v); 9.0 mL/min, 35°C, 100 bar, UV detection 245 nm.

While the analytical SFC condition produced adequate peak resolution in minimal run time, peak broadening was observed when the method was extended to semipreparative scale. Slight modifications were required for the preparative purification (20% modifier at a total flow of 9 mL/min). Under these conditions, 5 mg of (R,S)-goitrin could be injected at 3 min intervals using injection stacking (see Figure 4). The two enantiomers were collected as methanol dissolved solutes into separate pressurized collection vessels. Aliquots from the preparative collection vessels were removed and the individually isolated peaks were examined for chiral purity using the original analytical method; each was found to be greater than 99.0% pure by diode array detection. Optical rotation ([*α*]_*D*_^25^ (c, 1.0; chloroform)) of the separated (R)-goitrin and (S)-goitrin was +68.3° and −66.2°, respectively, which was close to the reported value [25]. The yield of the SFC preparation exceeded 90%, based on the total amount of the two isomers.

## 4. Conclusion

In summary, a novel analytical and preparative SFC separation method was developed for the separation of (R,S)-goitrin. Under optimized condition, (R)-goitrin and (S)-goitrin could be completely separated within 5 min, 10 times faster than the previous established NPLC method. Excellent precision, linearity, LOD, and LOQ were achieved. In addition, preparative resolution of the enantiomers with SFC showed unequivocal advantages in shorter time, easier removal of solvent, and much higher throughput. Finally, the separated material is now available in pure enantiomeric form for use in the pharmacodynamics comparison to illustrate the effective substance in Isatidis Radix.

## Figures and Tables

**Table 1:** Chromatographic data for separation of (R,S)-goitrin on Chiralpak IC column with different concentration of acetonitrile as cosolvent (column temperature: 35°C, flow rate: 3.5 mL/min, and back pressure: 100 bar).

Concentration (%)	Retention time (min)		Resolution
	S-goitrin	R-goitrin	
20	1.95	2.15	1.76
**15**	**3.04**	**3.48**	**3.78**
10	5.99	7.14	3.86
8	9.15	11.02	4.41
7	11.66	14.08	4.16

**Table 2 tab2:** Chromatographic data for separation of (R,S)-goitrin on Chiralpak IC column at different column temperature (concentration of ACN: 15%, flow rate: 3.5 mL/min, and back pressure: 100 bar).

Temperature (°C)	Retention time (min)		Resolution
	S-goitrin	R-goitrin	
**35**	**3.03**	**3.50**	**3.74**
40	2.96	3.43	3.32
45	3.21	3.75	3.05

**Table 3 tab3:** Chromatographic data for separation of (R,S)-goitrin on Chiralpak IC column with different flow rate (column temperature: 35°C, concentration of ACN: 15%, and back pressure: 100 bar).

Flow rate (mL/min)	Retention time (min)		Resolution
	S-goitrin	R-goitrin	
2.0	4.81	5.42	2.68
2.5	3.95	4.62	3.36
3.0	3.27	3.77	3.53
**3.5**	**3.02**	**3.55**	**3.70**
4.0	2.67	3.14	3.53

**Table 4:** Chromatographic data for separation of (R,S)-goitrin on Chiralpak IC column under different back pressure (column temperature: 35°C, concentration of ACN: 15%, and flow rate: 3.5 mL/min).

Back pressure (bar)	Retention time (min)		Resolution
	S-goitrin	R-goitrin	
**100**	**3.02**	**3.58**	**3.75**
120	2.97	3.51	3.48
150	2.90	3.42	3.37
180	2.84	3.34	3.33
200	2.82	3.31	3.30

**Table 5:** Linearity, precision, LOD, and LOQ.

	S-goitrin			R-goitrin		
Linearity range	0.0003, 0.01, 0.1, 1.0, 2.0					
Precision						
Concentration level (mg/mL)	0.0003	0.1	2.0	0.0003	0.1	2.0
Intraday (*n* = 3)	1.35%	0.41%	0.57%	1.21%	0.69%	0.77%
Interday (*n* = 3)	2.49%	1.62%	1.89%	2.67%	1.71%	1.49%
LOD (ng/mL)		91			97	
LOQ (ng/mL)		279			313	
